# Genomic Tools for Evolution and Conservation in the Chimpanzee: *Pan troglodytes ellioti* Is a Genetically Distinct Population

**DOI:** 10.1371/journal.pgen.1002504

**Published:** 2012-03-01

**Authors:** Rory Bowden, Tammie S. MacFie, Simon Myers, Garrett Hellenthal, Eric Nerrienet, Ronald E. Bontrop, Colin Freeman, Peter Donnelly, Nicholas I. Mundy

**Affiliations:** 1 Department of Statistics, University of Oxford, Oxford, United Kingdom; 2 Wellcome Trust Centre for Human Genetics, University of Oxford, Oxford, United Kingdom; 3 Department of Zoology, University of Cambridge, Cambridge, United Kingdom; 4 Broad Institute, Boston, Massachusetts, United States of America; 5 Centre Pasteur du Cameroun, Yaoundé, Cameroon; 6 Biomedical Primate Research Center, Rijswijk, The Netherlands; University of Arizona, United States of America

## Abstract

In spite of its evolutionary significance and conservation importance, the population structure of the common chimpanzee, *Pan troglodytes*, is still poorly understood. An issue of particular controversy is whether the proposed fourth subspecies of chimpanzee, *Pan troglodytes ellioti*, from parts of Nigeria and Cameroon, is genetically distinct. Although modern high-throughput SNP genotyping has had a major impact on our understanding of human population structure and demographic history, its application to ecological, demographic, or conservation questions in non-human species has been extremely limited. Here we apply these tools to chimpanzee population structure, using ∼700 autosomal SNPs derived from chimpanzee genomic data and a further ∼100 SNPs from targeted re-sequencing. We demonstrate conclusively the existence of *P. t. ellioti* as a genetically distinct subgroup. We show that there is clear differentiation between the *verus*, *troglodytes*, and *ellioti* populations at the SNP and haplotype level, on a scale that is greater than that separating continental human populations. Further, we show that only a small set of SNPs (10–20) is needed to successfully assign individuals to these populations. Tellingly, use of only mitochondrial DNA variation to classify individuals is erroneous in 4 of 54 cases, reinforcing the dangers of basing demographic inference on a single locus and implying that the demographic history of the species is more complicated than that suggested analyses based solely on mtDNA. In this study we demonstrate the feasibility of developing economical and robust tests of individual chimpanzee origin as well as in-depth studies of population structure. These findings have important implications for conservation strategies and our understanding of the evolution of chimpanzees. They also act as a proof-of-principle for the use of cheap high-throughput genomic methods for ecological questions.

## Author Summary

Chimpanzees are viewed with fondness as our closest animal relatives and are valued by scientists for the biological and evolutionary insights they provide. In spite of this, the relationships between different populations of common chimpanzees are still relatively poorly understood, a situation that potentially threatens conservation efforts. Here we have used information gathered in the Chimpanzee Genome Project to design comprehensive tests of genetic variability that show unambiguously the existence of four genetically distinct groups (or populations) of common chimpanzee. We demonstrate that previous methods based on mitochondrial DNA sequences alone are not always accurate and show the feasibility of cheap new genetic tests of individuals' origins that could play an important role in conservation.

## Introduction

**Figure 1:**
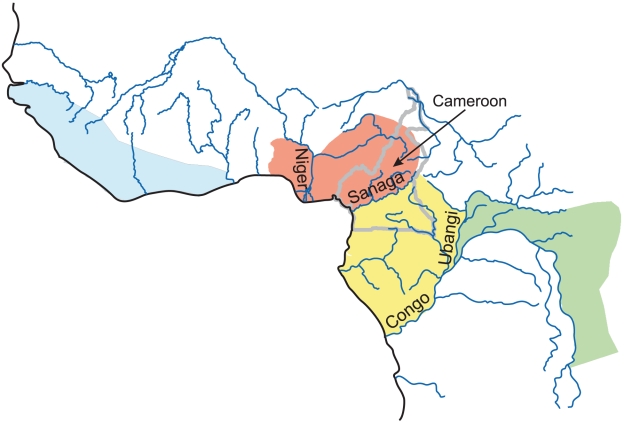
Map of the geographic distribution of four populations of common chimpanzee. *After* [*3*] *, Figure 6b*. Colours show the ranges of each population (yellow - *P. t. troglodytes*, red - *P. t. ellioti*, blue - *P. t. verus*, green - *P. t. schweinfurthii*) with major rivers indicated. The Sanaga River in Cameroon has been proposed to form the boundary between the ranges of *P. t. ellioti* and *P. t. troglodytes*.

The history and population structure of the common chimpanzee, *Pan troglodytes*, are incompletely understood. Traditionally, three subspecies have been described: the western chimpanzee (*P. t. verus*), central chimpanzee (*P. t. troglodytes*) and eastern chimpanzee (*P. t. schweinfurthii*). Analysis of mitochondrial DNA (mtDNA) variation led to the proposal of a fourth, “Nigerian” chimpanzee subspecies (*P. t. vellerosus*, since renamed *P. t. ellioti* [*1*]) as a sister taxon to *P. t. verus* occurring in an area of Nigeria and Cameroon east of the Niger river and north of the Sanaga river (Figure 1) [2], [3]. This new subspecies has been recognized by many taxonomists and conservation biologists [4], [5]. Subsequent analyses of autosomal microsatellite data, in one case based on few loci [6], and in another including few individuals designated *a priori* as *P. t. ellioti* [7], found little evidence to distinguish *P. t. ellioti* from *P. t. troglodytes*, which is distributed south of the Sanaga river (Figure 1). Very recently however a microsatellite-based study of 94 individuals with 27 loci [8] has established that up to five groups of common chimpanzees, including *P. t. ellioti*, can be distinguished genetically. In this study we provide a complementary analysis using very different data and analytical methodology that allows a direct comparison with human data.

For most animals, the definition of a subspecies as “a collection of populations occupying a distinct breeding range and diagnosably distinct from other populations” [9] would be uncontroversial. However, our close evolutionary relationship with chimpanzees, and the parallels that can be drawn between chimpanzees and humans, makes this terminology increasingly uncomfortable, and in some cases controversial, and so we prefer to avoid it. Whatever term is used, modern genetic methods clearly have the potential to make the assessment of distinctiveness more objective and precise than in the past and it should now be possible to confirm or refine earlier judgments that were based on other criteria or limited data.

The development of modern high-throughput SNP genotyping technologies has revolutionized many aspects of human genetics, including our understanding of the history and demography of human populations [10]–[13]. To date, the impact of such methods in non-human species has been limited (e.g. [14], [15]). Here we apply these technologies to chimpanzees, and show that they can clearly resolve the genetic distinctness of *P. t. ellioti*, and that, for conservation purposes, small subsets of SNPs can be used to distinguish previously recognized populations. Our major source of SNPs was those arising from sequencing reads of a single individual (“Clint”) from the chimpanzee genome project [16]. A notable finding is that, in spite of the severe ascertainment biases inherent in this SNP discovery (largely a single individual, from only one of the populations), analyses based on the resulting SNPs remain powerful, suggesting that the same may be true in other species for which there have been genome projects. We also demonstrate the potential benefits of haplotype-based analyses in combination with genomic SNP data in defining and quantifying population relationships.

## Results

To address the question of whether *Pan t. ellioti* is genetically distinct from other populations, we obtained DNA samples from Cameroonian chimpanzees which we analysed along with samples from captive Western (*verus*) and *troglodytes* chimpanzees. Eastern chimpanzees (*P. t. schweinfurthii*), with their distinct geographical distribution, were not sampled in the current study. We sequenced 12 autosomal fragments of ∼1 kb and genotyped 691 SNPs from 22 autosomal regions of 40–80 kb [17] in order to resolve genome-wide relationships, and compared the results with inference from the mitochondrial *HV-I* locus.

We applied a number of different methods to the analysis of these data to assess the relationships and genetic clustering amongst the sampled individuals. The first set of methods (principal components and Structure) were based on the marginal data at each genotyped SNP. We then calculated F_ST_ from the DNA sequence data, and finally applied recently developed methods which exploited information on the joint distribution of SNP alleles within haplotypes.

**Figure 2:**
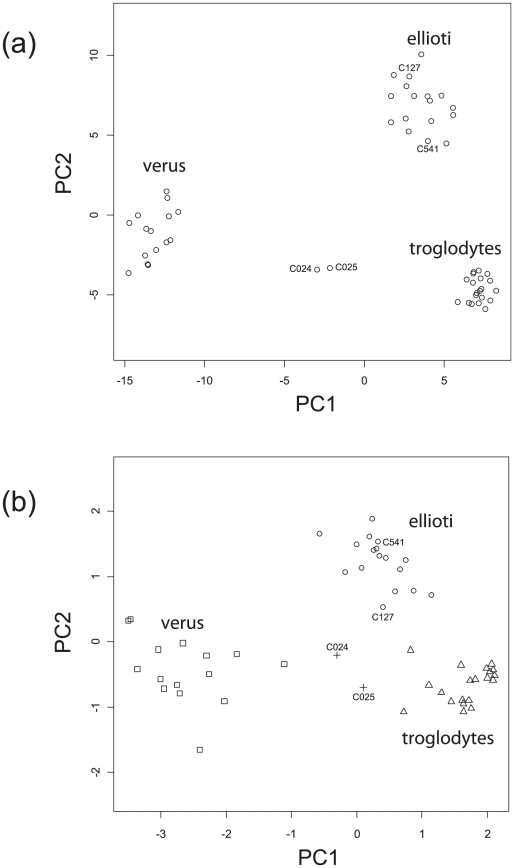
Clustering of chimpanzees based on principal components. (a) Clustering of chimpanzees based on principal components using data from 818 SNPs. Plots of the first two principal components of data from 818 SNPs show that chimpanzees in this study form three genetically distinct groups. Two chimpanzees (C127, C541) have *P. t. troglodytes*-like mtDNA but group with *P. t. ellioti* at autosomal loci. Two chimpanzees (C024, C025) known to be hybrids between *P. t. troglodytes* and *P. t. verus* lie between these populations on the PCA plot. (b) Clustering of chimpanzees based on principal components using population-informative SNPs. Plots of the first two principal components of data from just 10 selected SNPs (Table S4) reveal the same three groups as the full dataset. Plotted positions are shown with jitter to separate individuals with the same genotypes at the subset of SNPs. Plotting characters show the inferred population of origin of each chimpanzee: (triangles - *P. t. troglodytes*, squares - *P. t. ellioti*, circles - *P. t. verus*, ‘+’ - hybrids).

Using the first two principal components of the data from all 818 SNPs, 52 of the 54 chimpanzees studied clustered into three distinct, non-overlapping groups (Figure 2a). These clusters are consistent with three genetically distinct populations represented amongst the study chimpanzees: captive Western (*P. t. verus*) chimpanzees form one cluster while Cameroonian chimpanzees are divided into two genetically distinct clusters, one of which we infer to correspond to *P. t. ellioti*, whose existence had been the subject of uncertainty. We note that two individuals in the *P. t. ellioti* cluster had previously been designated *P. t. troglodytes* based on mtDNA sequence, a point to which we return below. Two individuals (C024, C025) with *P. t. troglodytes*-like mtDNA lie between the presumptive *P. t. verus* and *P. t. troglodytes* clusters, and records have subsequently revealed that these are indeed first-generation hybrids produced in captivity.

**Figure 3:**
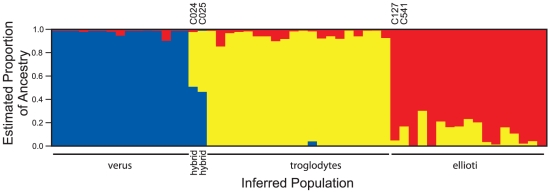
Structure estimates of ancestry in three populations. For each sampled individual the figure shows the estimated proportion of ancestry from Structure's three putative ancestral populations, with *P. t. troglodytes* in yellow, *P. t. ellioti* in red and *P. t. verus* in blue. Structure reveals the same pattern of group memberships as PCA, and additionally suggests that *P. t. troglodytes* and *P. t. ellioti* individuals may share more DNA from the other group than either shares with *P. t. verus* (blue). The two known hybrid individuals (C024, C025, with ancestry estimated at close to 50% in each of *P. t. troglodytes* and *P. t. verus*) and two *P. t. ellioti* chimpanzees with *P. t. troglodytes*-like mtDNA (C127, C541) are labelled.

A similar conclusion comes from a different perspective when the software Structure [18], [19] is used to estimate the proportion of each individual's genome that comes from each of several ancestral populations. With k = 3 presumptive populations, the same three groups were recovered cleanly with little estimated admixture except for the two hybrids (Figure 3), and where there was evidence for co-ancestry, it was detected between the *ellioti* and *troglodytes* groups, rather than involving *verus* chimpanzees. This suggests more recent interaction between *P. t. ellioti* and *P. t. troglodytes* than either has had with *P. t. verus*, although an effect of SNP ascertainment could not be ruled out. We note that the model underlying Structure assumes no linkage disequilibrium between loci, whereas our data do exhibit such correlations because of the clustering of SNPs. The expected effect of this in the Structure model is an over-estimation of precision, rather than bias [19], but nonetheless our Structure analysis should be interpreted with some caution.

Next, we calculated pairwise F_ST_, a commonly-used measure of the proportion of total genetic variation occurring between populations. Potential confounding effects from SNP ascertainment complicate interpretation of F_ST_ values calculated from the genotype data, so we restricted these analyses to our re-sequencing data alone (104 of 818 SNPs, also eliminating 3 sequenced loci showing evidence of positive selection) [20]. Consistent with Structure's view of relative amounts of co-ancestry, F_ST_ between *P. t. ellioti and P. t. troglodytes* (0.134, 95% CI 0.105–0.162) is slightly lower than, but cannot be formally distinguished from, that between *P. t. troglodytes* and *P. t. verus* (0.177, 95% CI 0.129–0.225) or between *P. t. ellioti* and *P. t. verus* (0.190, 95% CI 0.145–0.235). The *troglodytes* – *verus* figure in our data is lower than the 0.29 for Central vs. Western chimpanzees previously estimated from re-sequencing data [21], presumably due to sampling differences (either of loci or individuals) between the two studies.

When genetic data is collected from tightly linked variable sites, exploiting patterns of non-random association (i.e. linkage disequilibrium) can increase power to identify population structure over single-SNP analyses [22], [23]. Informally, haplotype-based approaches have many of the advantages in terms of discriminatory power of other multi-allelic systems such as microsatellites, but in addition, our understanding of the evolutionary mechanisms involved means that there is a natural sense of the evolutionary distance between haplotypes. Sensible haplotype-based analyses can thus be more powerful than SNP-based approaches in using considerably more genetic information in comparing individuals, and in our context can thus be informative about differentiation at timescales shorter than those over which drift can be detected in SNP frequency differences. Additionally, haplotype-based analyses may be less susceptible to biases in SNP discovery [22]. Conversely, while haplotype-based methods can increase power to detect population structure, statistical methodology to fit explicit models of isolation, migration and fluctuating population size [24] to such data is so far lacking.

**Figure 4:**
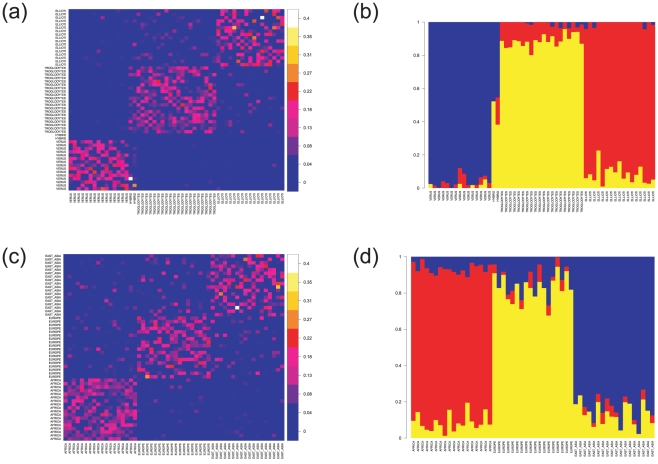
Haplotype-based analyses of population relationships. (a) (chimpanzee) and (c) (human): heat maps show the estimates from a copying model of the proportion of sampled genetic material of each individual (X axis) inferred to be closest to that in each other individual in the sample (Y axis). Human data was sampled from HapMap data for the three continental populations: Europe (CEU), Africa (YRI) and East Asia (Han Chinese, CHB) using an ascertainment scheme designed to match properties of SNPs in the chimpanzee data. Chimpanzees have less estimated copying from outside their own population than do humans. Individuals are labeled by their inferred (chimpanzee) or known (human) population of origin, or as hybrids. (b) and (d): summaries of estimated copying (ancestry) proportions by population, for each individual. (b) Chimpanzees: *P. t. troglodytes* in yellow, *P. t. ellioti* in red and *P. t. verus* in blue. *P. t. troglodytes* and *P. t. ellioti* appear to be less differentiated from other populations than is *P. t. verus*. (**d**) Human Continental populations: CEU Europe in yellow, YOR Africa in red and CHB East Asia in blue. Human individuals have higher proportions of ancestry from other populations than do chimpanzees.

We analysed similarities in patterns of haplotype variation among individuals for the 691 clustered autosomal SNPs using a so-called copying model applied to estimated haplotypes from each individual [25], [26]. In effect, for each small chromosomal segment in one of the haplotypes of a particular individual, the approach looks amongst the haplotypes of the other sampled individuals to find the one with which it is most closely related, in the sense of most recently sharing a common ancestor. This is done under a model in which shared ancestry is likely to be the same for chromosomal segments which are very near to each other (in terms of genetic distance). The primary results of such an analysis are estimates of the most recent shared ancestry across each locus in each haplotype. For a particular chimpanzee, these can be aggregated to calculate the estimated proportion of the sampled regions for which it is most closely related to each of the other chimpanzees. These estimates are shown in Figure 4a. The figure provides a visual summary of the patterns of most-recently-shared ancestry within and between the three population groups. In a randomly mating population, the haplotypes in a particular individual will share similarities with many others across the sample, while in the presence of population structure haplotypes will tend to be more similar to those of other individuals within the same population than to those in other populations. Figure 4b (see also Table 1) provides a higher-level summary which aggregates information across populations to show, for each chimpanzee, the proportion of its sampled regions for which the most closely related haplotype comes from each of the three populations. Strikingly, Figure 4a and 4b show that across most of the sampled regions in each individual, the most closely related haplotype comes from the same population; in other words that the three populations are genetically quite distinct. This effect is most marked for the *P. t. verus* individuals, for whom the most closely related haplotype is virtually always in the same population. Haplotypes of *P. t. ellioti* and *P. t. troglodytes* chimpanzees respectively are typically most similar to those of other individuals within the same population, but occasionally to those of individuals from the other (*P. t. troglodytes* and *P. t. ellioti* respectively) population. The two previously noted hybrid individuals are clearly identified, and in addition it emerges that two of the *P. t. ellioti* chimpanzees had a higher level of shared ancestry than the other chimpanzees. The qualitative conclusions from the haplotype-based analysis thus mimic those from principal components and Structure, although reassuringly they explicitly model the correlations between nearby SNPs, in contrast to Structure.

**Table 1:** Estimates of the proportion of the sampled genomic regions for which the most closely related haplotype comes from each study population, for chimpanzees and humans.

Chimpanzees				
	copying population	copying from		
		*P. t. troglodytes*	*P. t. ellioti*	*P. t. verus*
	***P. t. troglodytes***	0.887	0.105	0.009
	***P. t. ellioti***	0.084	0.908	0.008
	***P. t. verus***	0.009	0.028	0.962

Humans				
	copying population	copying from		
		CEU Europe	YOR Africa	CHB East Asia
	**CEU Europe**	0.837 (0.791–0.870)	0.036 (0.022–0.051)	0.127 (0.095–0.169)
	**YOR Africa**	0.080 (0.055–0.104)	0.860 (0.834–0.892)	0.060 (0.035–0.080)
	**CHB East Asia**	0.125 (0.089–0.174)	0.023 (0.010–0.032)	0.852 (0.807–0.888)

Parentheses show the empirical central 95% region of the distribution of values for the 100 re-samples of the human data.

By applying the haplotype-based copying model to human data, we can compare quantitatively the extent of differentiation between the three chimpanzee groups with that between various human populations. Importantly, such analyses can allow for ascertainment effects. We show the copy model results for human data from the Phase II HapMap (Frazer et al. 2007) in Figure 4c and 4d, comparing sampled individuals of European (CEPH), African (Yoruba, YRI) and East-Asian (Han Chinese, CHB) descent in an analysis in which SNPs in the human data were re-ascertained to match characteristics of the chimpanzee data (see “Data Analysis”). The average within- vs. between-population copying frequencies, that is, frequencies for the most-closely-related-haplotype, in these analyses are summarized in Table 1. Levels of between-population similarity among the chimpanzee populations are lower than among the HapMap populations, suggesting that the chimpanzee populations are more distinct than even continental human populations. To test the robustness of this conclusion to choice of comparison data, we re-sampled Phase II HapMap individuals, genomic regions and ascertained SNPs, 100 times. Only three times was the level of within-population copying of a pair of human populations greater than that between any chimp population (estimated within-population copying in each of Africa and East-Asia was greater than the estimated within-population copying in *P. t. troglodytes* for 3 of 100 re-samples). In Figures S1 and S2, we colour fragments of chromosomes according to their assigned population of origin under the copying model, illustrating that the probabilities with which individual chimpanzee chromosome segments are assigned to specific populations are also higher than for human data.

An equivalent analysis of the HapMap III African populations [27] showed that these African human populations are considerably less structured than the chimpanzee populations (Figures S3 and S4), as might be expected given the observation above that the chimpanzee populations are more differentiated even than continental human populations. Note that our comparisons with the human population samples are based on similar amounts of data as in our chimpanzee samples. With larger SNP datasets, the power to separate the human populations increases.

We note that while it is theoretically possible to use the lengths of copied fragments in the copying model to estimate the timescale over which differentiation has occurred, our data is not well-suited to this because the shortness of the assayed regions means that relatively few breakpoints are observed, providing little information about the times of events in the history of chimpanzee populations.

## Discussion

We have applied a number of different analytical methods to an extensive set of SNP data from 54 chimpanzees. All of the methods point clearly to the existence of three distinct population groups, corresponding to three of the previously-described “subspecies” of chimpanzee *P. t. verus*, *P. t. troglodytes*, and *P. t. ellioti*, with the latter two groups sharing somewhat more similarity with each other than either does with *P. t. verus*. *P. t. troglodytes and P. t. verus* are two securely defined populations estimated to have diverged 0.4–0.6 million years ago [7], [8], [28]–[30]. Our analyses show *P. t. ellioti* to be clearly distinct from *P. t. troglodytes* with both groups equally distinct from *P. t. verus*, so that whatever terminology (“population” or “subspecies”) is applied to *verus* and *troglodytes* should equally be applied to *ellioti*.

By way of comparison, we have shown that these three chimpanzee populations are more differentiated than even continental human populations, and also that in spite of the relatively close geographic proximity of the groups, particularly *troglodytes* and *ellioti*, the chimpanzee populations are considerably more distinct than the African populations sampled in HapMap III, suggesting rather differing demographic histories for the two sister species.

In order to compare population comparisons based on the copying model with those based on more traditional F_ST_ approaches, we also calculated pairwise F_ST_ values for each of the 100 resamples of individuals and SNPs in our analyses of the three continental population samples. The results are summarized in Table 2. We note that while the average values of pairwise F_ST_ across the 100 samples show the same pattern as copying proportions in the copying model, the sample-to-sample variation is larger. For example, the F_ST_ intervals for the central 95% of resamples for Europe-East Asia overlap those of Africa-Europe and Africa-East Asia, and for example for five of the 100 resamples the pairwise F_ST_ between Africa and Europe was actually smaller than that between Europe and East-Asia. In contrast, for the copying model analysis the 95% intervals for the proportion that Europe and East Asia copy from each other do not overlap with the 95% intervals for either copying from Africa, and the proportion that Europe copied from Africa was lower than the proportion Europe copied from East Asia in each of the 100 re-samples. This accurately reflects the fact that on average East Asia and Europe share more recent ancestry with each other than with Africa.

**Table 2:** Pairwise F_ST_ values for human samples.

	CEU Europe	YOR Africa	CHB East Asia
**CEU Europe**	-	0.150 (0.120–0.190)	0.108 (0.075–0.145)
**YOR Africa**	0.150 (0.120–0.190)	-	0.172 (0.133–0.223)
**CHB East Asia**	0.108 (0.075–0.145)	0.172 (0.133–0.223)	-

Parentheses show the empirical central 95% region of the distribution of values for the 100 re-samples of the human data.

One weakness of our study (and some others) is that we do not have definitive information on the geographic origin of all of the chimpanzees we have studied. All our analyses point to two very distinct population groups for the chimpanzees originating from eastern Nigeria and Cameroon. In the light of other genetic evidence for distinctiveness of individuals sampled from either side of the Sanaga River [3], [8], our assignment of one of our sampled groups as *troglodytes* and one as *ellioti* seems reasonable. Whilst our data alone could not rule out two distinct populations, one or both of which extends across the Sanaga River, this seems *a priori* unlikely – the river provides a natural barrier between the distinct populations, whereas if both were to exist on the same side of the river there seems no reason for their reproductive isolation—and at variance to other available evidence. Notwithstanding our lack of complete geographical information on sampled chimpanzees, the clear separation between all three populations, relative to the similarities within the populations, seems hard to reconcile with the suggestion that chimpanzee genetic variation is distributed more or less continuously across the species range (cf [21]).

The initial genetic description of *P. t. ellioti* was based on mtDNA sequence analysis [2], [3], which places most chimpanzees from parts of Nigeria and Cameroon north of the Sanaga river in a group sharing a common ancestor with *P. t. verus*, to the exclusion of *P. t. troglodytes*, a description made more robust by a recent analysis of complete mitochondrial genomes [31], [32]. We compared the classification based on mtDNA with our genome-wide analysis and found that it classified 50 of 52 non-hybrid individuals correctly. Chimpanzees C127 and C541 had *troglodytes*-like mtDNA but *ellioti* autosomal SNP genotypes. (The two known hybrid chimpanzees C024 and C025 had *troglodytes*-like mtDNA but were detectably intermediate in autosomal genotype). Thus the two systems generally agree, but, not surprisingly, single-locus mtDNA data is less reliable for classification than genome-wide data.

The mtDNA-based picture of demographic relationships suggests that *P. t. verus* and *P. t. ellioti* are sister taxa [3], [31]. Our data suggests this to be misleading, in two different respects. Firstly, as noted above, two individuals who are clearly *P. t. ellioti*, on the basis of extensive autosomal data, have mtDNA which clusters with *P. t. troglodytes*. Thus, mtDNA from *ellioti* individuals does not fall into a single clade on a mtDNA tree. If mtDNA is used both to classify individuals and to estimate trees for the resulting groups, there is always a danger, as seems to have occurred in this instance, that misclassification of individuals will lead to a simpler-looking tree than is actually the case. Secondly, the suggestion from the mtDNA data that (many, but as noted above, not all) *ellioti* individuals have mtDNA types which are closer to *verus* than to *troglodytes* individuals is strikingly different from the results of our analyses based on many independent autosomal loci, which places *P. t. ellioti* clearly closer to *P. t. troglodytes* than to *P. t. verus*. It is interesting to note that a study of morphological variation agreed with the picture obtained from autosomal loci [4]. Taken together, the mtDNA and autosomal results are difficult to reconcile with a simple demographic scenario based on population splitting, and suggest a more complex demographic history for the three populations we have studied, possibly including sex-biased gene flow.

For many conservation applications, it would be desirable to be able to assign or classify individuals to populations based on a small number of loci. We developed and applied a method for choosing subsets of SNPs for classification based on their contribution to assignment probabilities (see Methods). To avoid over-fitting, we divided our data set in two. A training dataset comprising half the samples from each population (27 of the 52 non-hybrid individuals) was used to select informative SNPs for classification, with the other half of the individuals forming a test dataset in which the ability of the chosen SNPs to accurately classify individuals to populations was measured.

For our data, we could essentially reproduce the discrimination obtained with the complete dataset of 818 SNPs with as few as 8 carefully selected SNPs in distinct regions of the genome (Figure 2b). While there is still some danger of over-fitting from our relatively small sample sizes, we conclude that a small, well-chosen panel of probably 10–20 SNPs, assayed via either a set of PCR-based single-locus assays or a single multiplex SNP assay for forensic and conservation work, would be capable of analysing and classifying limited DNA samples at low cost. The exact size of panel used would depend on the requirement to identify individuals of mixed ancestry. This is particularly encouraging considering the extreme ascertainment bias inherent in our genotyped SNPs: for the chimpanzee, dbSNP at the time of our SNP selection reflected the composition of the chimpanzee draft genome, in which ∼91% of sequence traces came from a single *P. t. verus* individual (‘Clint’), a further 4% from four other *verus*, and less than 5% from three *P. t. troglodytes* [16]. Notwithstanding this bias, 12 of our SNPs have an estimated allele frequency difference of >0.5 between *ellioti* and pooled *troglodytes* and *verus* chimpanzees. Our study thus confirms the utility of genomic resources even when ascertainment is sub-optimal.

The confirmation of *P. t. ellioti* as a genetically distinct population of chimpanzee strongly supports efforts to treat this population as a separate management unit for conservation [33] This is of particular importance since while all chimpanzees are considered to be endangered [34], *P. t. ellioti*, with an estimated 6,500 individuals remaining, is the least numerous population.

In conclusion, using genomic resources we have assembled the largest SNP-based dataset for investigating chimpanzee population structure. It resolves an outstanding controversy in clearly establishing the fourth putative subspecies, *Pan troglodytes ellioti*, as a genetically distinct group. More generally, our results confirm the utility of high throughput SNP typing for evolutionary genetic and conservation analysis. However, we recognize that a full appraisal of chimpanzee population structure would require denser sampling from all four populations in addition potentially to comparative studies across primates that go beyond great apes and humans.

## Materials and Methods

### Chimpanzee samples

Blood samples were obtained from 35 wild-born orphaned chimpanzees of unknown geographic origin within Cameroon. Genomic DNA, extracted using standard procedures, was amplified (GenomiPhi, GE Healthcare) before genotyping. DNA samples were also obtained from 15 *P. t. verus* (from Sierra Leone) and 4 putative *P. t. troglodytes* (unknown geographic origin) chimpanzees held at the Biomedical Primate Research Centre in the Netherlands (Table S1). For chimpanzees in the Netherlands, all blood sampling was done in accordance with a protocol that was approved by the Institutional Animal Care and User Committee (IACUC) of the Biomedical Primate Research Center (BPRC). For chimpanzees in Cameroon, blood samples were taken from orphaned individuals for haematological analysis as part of veterinary health screens.

### Re-sequencing

Mitochondrial HV-I fragments of 534 bp and fragments of ∼1 kbp from the genes *CCR5*, *SDF*, *CXCR4*, *CX3CR1*, *RANTES*, *CCR2*, *SEC22L3*, *ZNF445*, *PTPN23*, *CCRL2*, *MC1R* and *HBB* (Table S2, Table S3) were amplified by PCR and sequenced directly. PCR products with heterozygous indels were cloned and 10 clones were sequenced for each sample. For pairwise Fst analyses, 3 loci with evidence for directional selection (*CCR5*, *CXCR4* and *CX3CR1*; 23 SNPs, MacFie et al. 2009) were removed from the analysis.

### SNP genotyping

A panel of 768 SNPs was designed for the GoldenGate Genotyping Assay (Illumina, San Diego), using polymorphism information from the Chimpanzee Genome Project [16] via dbSNP v26 [http://www.ncbi.nlm.nih.gov/projects/SNP/]. The SNPs, arranged in 22 clusters of size 40–80 kbp on several autosomes, were screened using BLAST to ensure unique context. The panel has also been used to assess recombination rates in the 22 regions, orthologous to recombination hotspots in humans [17]. Across 54 samples, 58 SNPs failed visual inspection, 14 gave at least one no-call and 5 SNPs departed strongly from Hardy-Weinberg equilibrium within a population (as initially labelled), leaving 691 SNPs for analysis.

### Data analysis

Population structure was assessed by pairwise F_ST_ in Arlequin (with 95% CIs estimated by jackknifing) [35], PCA and SNP selection for assignment in the R Package [36], and with Structure [18], [19], using the admixture model of ancestry, with correlated allele frequencies, run with a ‘burn-in’ of 100,000 iterations followed by a further 1,000,000 iterations. This model is not strictly applicable to data from sites in linkage disequilibrium, so this analysis is indicative only. SNPs were chosen for classification as follows: for each SNP a sample was assigned to the population in which its genotype was most probable, the 818 SNPs were ranked by their ability to classify the training samples and the best SNPs, from distinct loci, were chosen (Table S4).

For the haplotype-based analysis, we inferred haplotypes and population-scaled recombination rates between adjacent SNPs using PHASEv2.1.1 [25], [37]–[39] with ten times the default number of MCMC iterations. We then applied the Li and Stephens (2003) copying model to the inferred “best-guess” haplotypes as described in [40] but fixing the PHASE recombination rate estimates, inferring the expected number of haplotype segments that each chimp copies from every other chimp via 100 iterations of an Expectation-Maximization (EM) algorithm and precluding copying from the other haplotype within the same individual. Figures S1, S2, and S3 are based on 100 samples from the model using the converged E-M values.

For comparisons with human data, we matched features of the chimpanzee dataset by randomly selecting 18 individuals per population using HapMap Phase 2 Release 21 or HapMap Phase 3 Release 2 consensus haplotypes. For each analysis, we then randomly selected 22 autosomal genomic regions, randomly selecting SNPs to match the SNP density and minor allele frequency distribution (in bins of (0.0,0.1], (0.1,0.2], (0.2,0.3], (0.3,0.4], (0.4,0.5]) for the respective 22 chimp regions. We ran the copying model using fixed genetic map estimates (build 35 estimates for HapMap2 populations and build 36 estimates for HapMap3 populations) scaled by an effective population size value of 30000, the value that maximized the expected log-likelihood over a fixed grid of (10K,20K,30K,40K,60K,300K,25000K), though we note that results were similar for all scaling factors we considered. Ascertaining SNPs on a single randomly selected HapMap Phase2 CEPH individual or HapMap Phase3 Luhya (Kenya) individual not included in the sample gave similar results to those presented. Pairwise F_ST_ for each re-sample was calculated using the approach described in [41].

## Supporting Information

Figure S1 Assignment of population of origin by genomic fragment: Chimpanzee Data. Each line in the figure shows an individual with its inferred population of origin and 22 autosomal fragments for which SNP genotype data was collected. Each line is divided into two coloured strips showing the two haplotypes for each fragment. Colours show the copying model-estimated probabilities of origin of each fragment for each chromosome (yellow - *P. t. troglodytes*, red - *P. t. ellioti*, blue - *P. t. verus*) and intermediate colours show intermediate probabilities. Chimpanzees have individual- and fragment-based copying probabilities that are more extreme (closer to 0 or 1) than human Continental populations, indicating greater population differentiation.
(PDF)

Figure S2 Assignment of population of origin by chromosomal fragment: Human Data. Figure as in Figure S1 for human continental population data sampled from HapMap data. Colours are yellow – CEU Europe, red – YOR Africa, blue – CHB East Asian. Human continental populations are much less differentiated at the individual and fragment level than chimpanzees.
(PDF)

Figure S3 Assignment of population of origin by chromosomal fragment: African Populations Data. Figure as in Figuress S1 and S2 for human African population data sampled from HapMap data. Colours are yellow – Luhya, red – Maasai, blue – Yoruba. Population differentiation is much less clear than for continental human or chimpanzee populations.
(PDF)

Figure S4 Haplotype-based analyses of population relationships. Figures as in Main Figure 4 for human African population data sampled from HapMap data. (a) heat map of estimated proportion of each individual (X axis) with most recent common ancestry with each other individual in the sample (Y axis); (b) estimated copying (ancestry) proportions by population, for each individual. Colours are yellow – Luhya, red – Maasai, blue – Yoruba. Population differentiation is much less clear than for continental human or chimpanzee populations.
(PDF)

Table S1 Chimpanzees Studied. BPRC = Biomedical Primate Research Centre, The Netherlands. mtDNA classification: T, *P. t. troglodytes*; E, *P. t. ellioti*; W, ‘Western’ i.e. *P. t. verus*.
(DOC)

Table S2 Amplification Targets.
(DOC)

Table S3 PCR and Sequencing Primers. PCR primers are in bold. All primers were used for sequencing.
(DOC)

Table S4 Highly Differentiated SNPs. For each population, SNPs with highest frequency difference between chimpanzees in that population cluster and the other two clusters. Bold SNPs are in the minimal panel of 10 markers used in Figure 2b to reproduce the original clustering pattern; SNPs from re-sequencing are underlined.
(DOC)
