# Supplementary figures and images for: Genomic Tools for Evolution and Conservation in the Chimpanzee: Pan troglodytes ellioti Is a Genetically Distinct Population
Source: PLoS Genet. 2012 Mar 1;8(3):e1002504. doi: 10.1371/journal.pgen.1002504 (PMC3291532; doi:10.1371/journal.pgen.1002504)

Fig S1

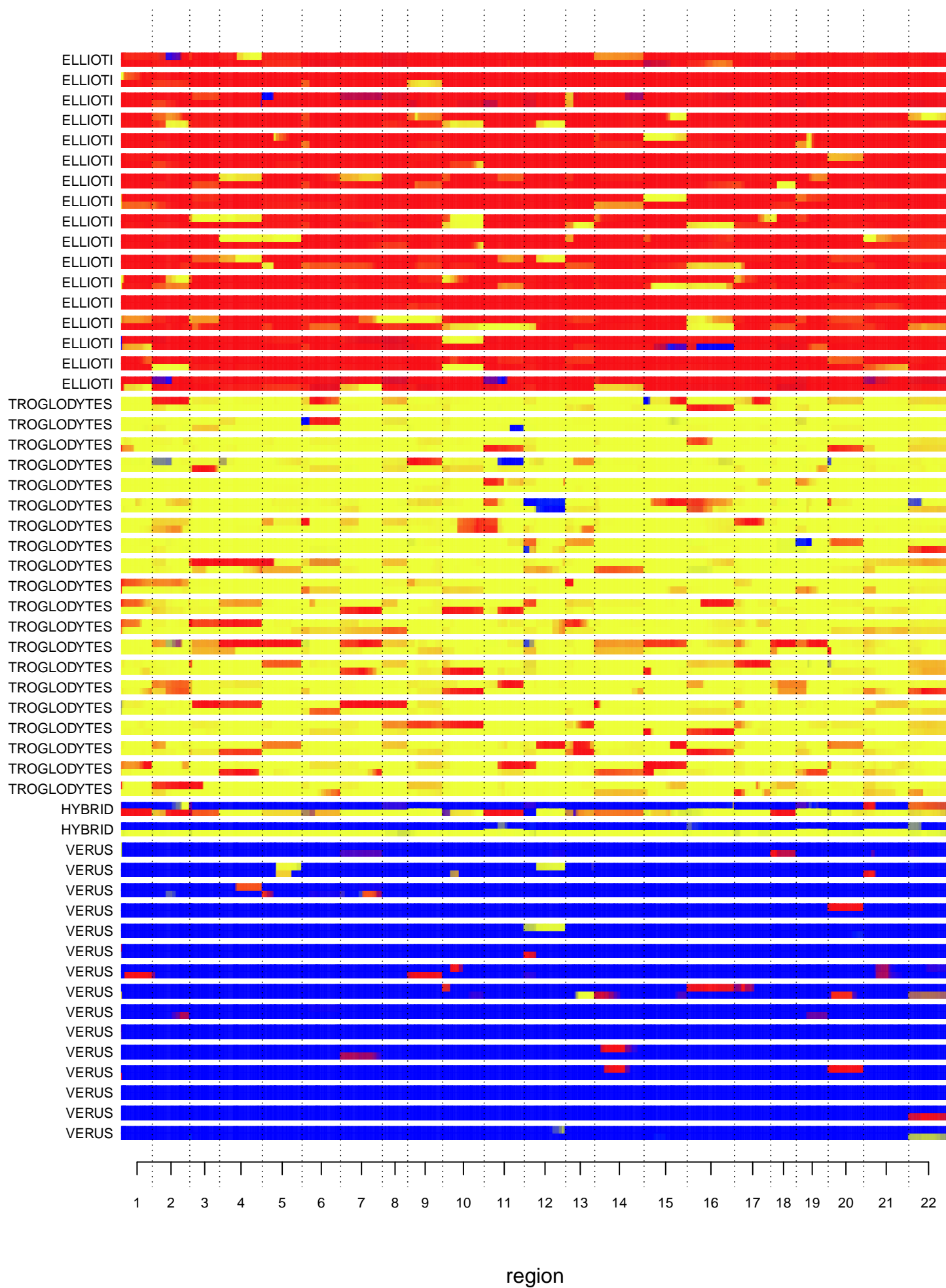

Supplement: Figure S1 — Assignment of population of origin by genomic fragment: Chimpanzee Data. Each line in the figure shows an individual with its inferred population of origin and 22 autosomal fragments for which SNP genotype data was collected. Each line is divided into two coloured strips showing the two haplotypes for each fragment. Colours show the copying model-estimated probabilities of origin of each fragment for each chromosome (yellow - P. t. troglodytes, red - P. t. ellioti, blue - P. t. verus) and intermediate colours show intermediate probabilities. Chimpanzees have individual- and fragment-based copying probabilities that are more extreme (closer to 0 or 1) than human Continental populations, indicating greater population differentiation. (PDF) [file pgen.1002504.s001.pdf]

Fig S2

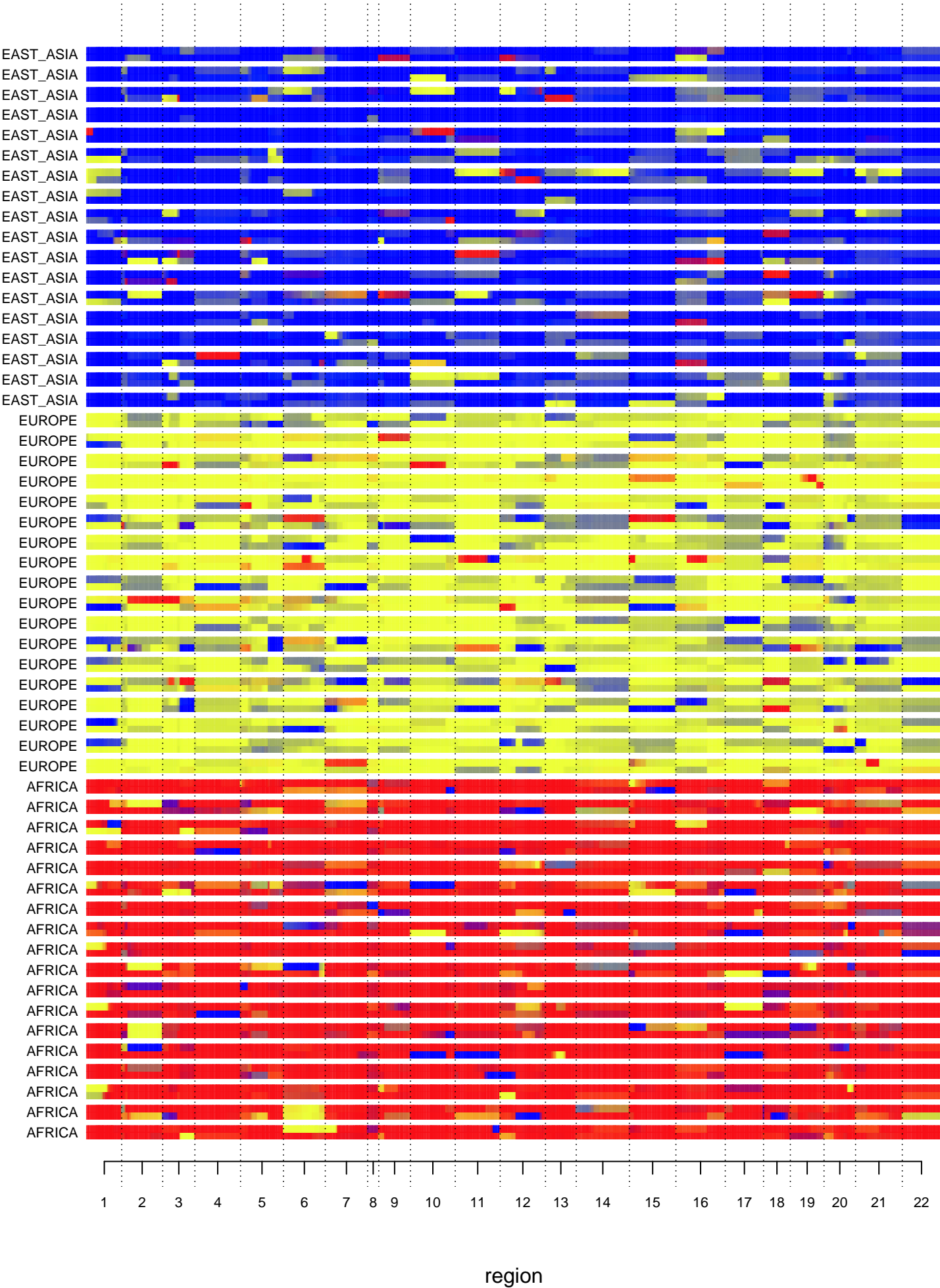

Supplement: Figure S2 — Assignment of population of origin by chromosomal fragment: Human Data. Figure as in Figure S1 for human continental population data sampled from HapMap data. Colours are yellow – CEU Europe, red – YOR Africa, blue – CHB East Asian. Human continental populations are much less differentiated at the individual and fragment level than chimpanzees. (PDF) [file pgen.1002504.s002.pdf]

Fig S3

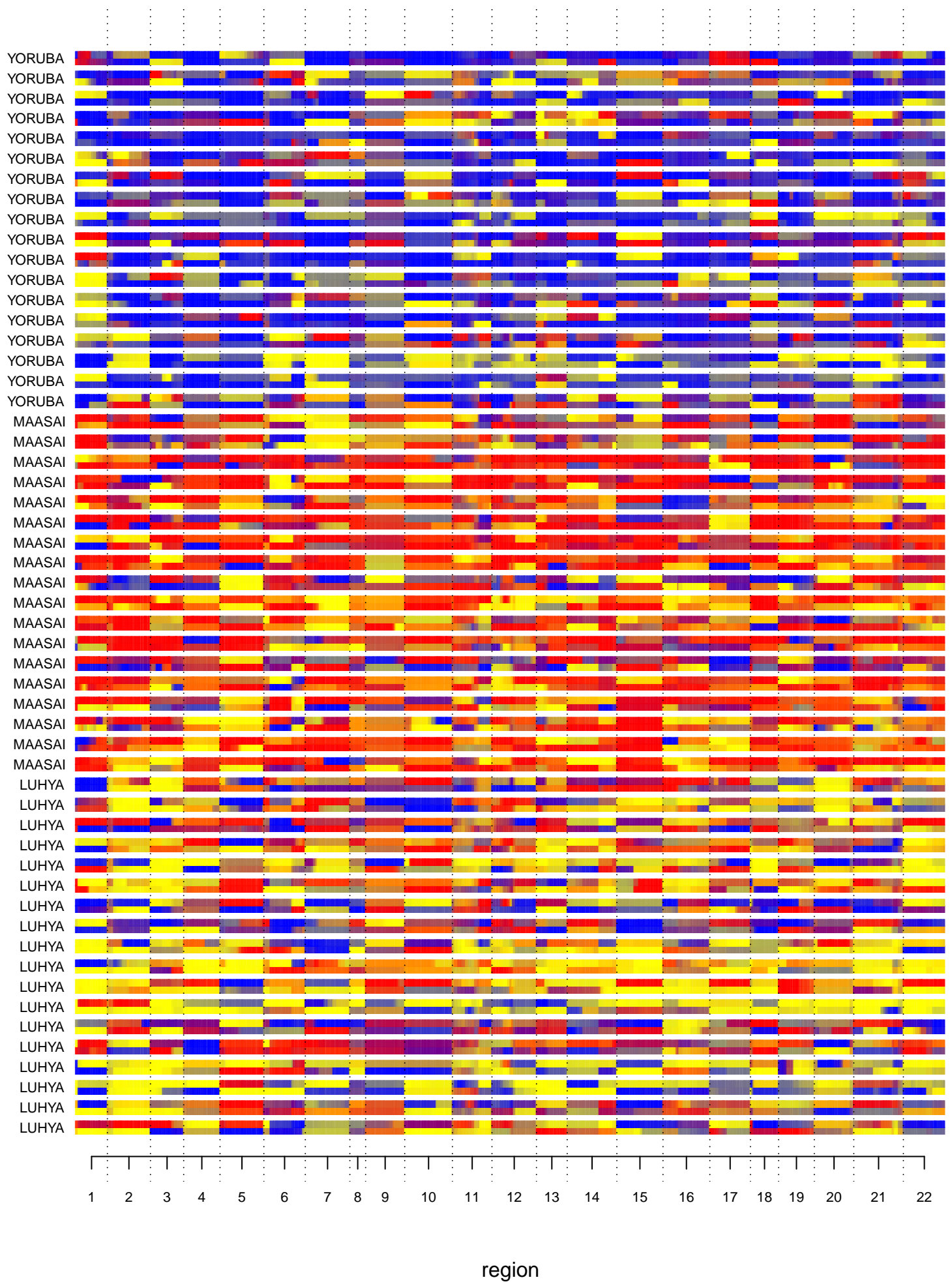

Supplement: Figure S3 — Assignment of population of origin by chromosomal fragment: African Populations Data. Figure as in Figuress S1 and S2 for human African population data sampled from HapMap data. Colours are yellow – Luhya, red – Maasai, blue – Yoruba. Population differentiation is much less clear than for continental human or chimpanzee populations. (PDF) [file pgen.1002504.s003.pdf]

Fig S4 (a)

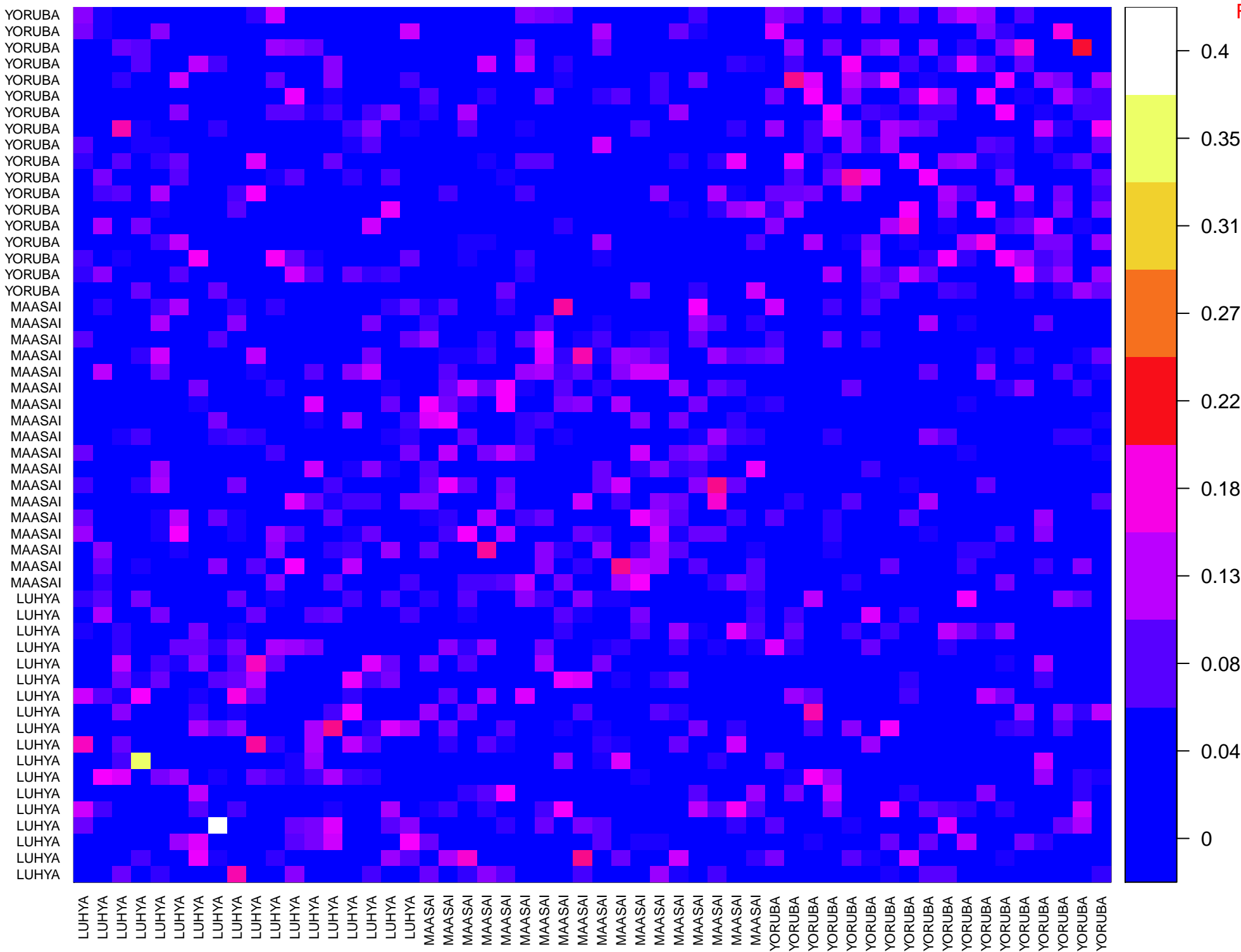

Fig S4 (b)

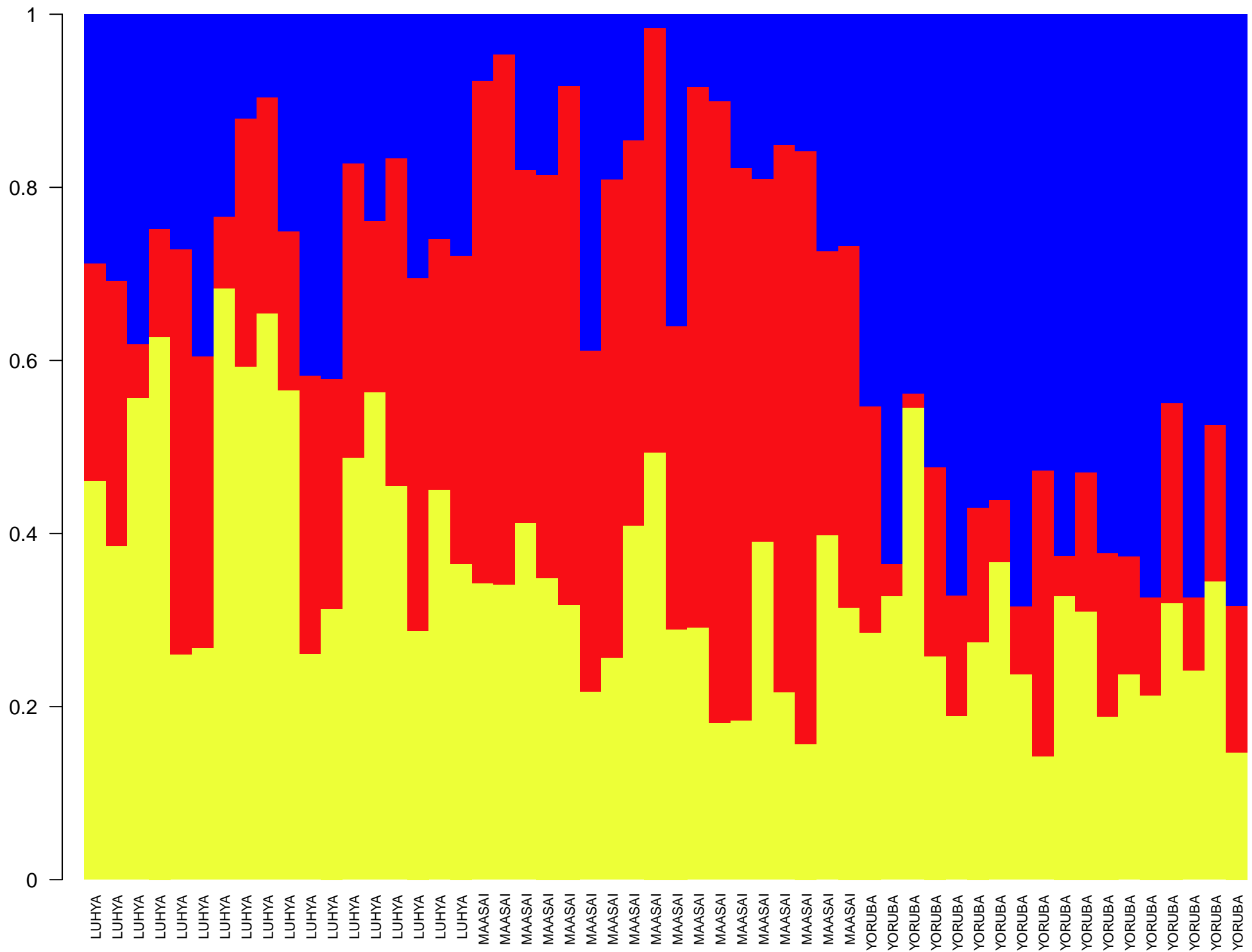

Supplement: Figure S4 — Haplotype-based analyses of population relationships. Figures as in Main Figure 4 for human African population data sampled from HapMap data. (a) heat map of estimated proportion of each individual (X axis) with most recent common ancestry with each other individual in the sample (Y axis); (b) estimated copying (ancestry) proportions by population, for each individual. Colours are yellow – Luhya, red – Maasai, blue – Yoruba. Population differentiation is much less clear than for continental human or chimpanzee populations. (PDF) [file pgen.1002504.s004.pdf]
