# Supplementary material for: Genomic Tools for Evolution and Conservation in the Chimpanzee: Pan troglodytes ellioti Is a Genetically Distinct Population
Source: PLoS Genet. 2012 Mar 1;8(3):e1002504. doi: 10.1371/journal.pgen.1002504 (PMC3291532; doi:10.1371/journal.pgen.1002504)
Supplement: Table S1 — Chimpanzees Studied. BPRC = Biomedical Primate Research Centre, The Netherlands. mtDNA classification: T, P. t. troglodytes; E, P. t. ellioti; W, ‘Western’ i.e. P. t. verus. (DOC) [file pgen.1002504.s005.doc]

### Table S1: Chimpanzees Studied

| origin | identifier | mtDNA classification |
| --- | --- | --- |
| Cameroon | C011 | T |
| Cameroon | C012 | E |
| Cameroon | C022 | T |
| Cameroon | C023 | T |
| Cameroon | C024 | T |
| Cameroon | C025 | T |
| Cameroon | C059 | E |
| Cameroon | C080 | T |
| Cameroon | C090 | E |
| Cameroon | C127 | T |
| Cameroon | C128 | T |
| Cameroon | C129 | T |
| Cameroon | C199 | T |
| Cameroon | C200 | E |
| Cameroon | C209 | T |
| Cameroon | C225 | T |
| Cameroon | C226 | T |
| Cameroon | C227 | T |
| Cameroon | C230 | T |
| Cameroon | C234 | E |
| Cameroon | C236 | T |
| Cameroon | C237 | E |
| Cameroon | C241 | E |
| Cameroon | C243 | T |
| Cameroon | C252 | E |
| Cameroon | C253 | E |
| Cameroon | C255 | E |
| Cameroon | C266 | T |
| Cameroon | C276 | T |
| Cameroon | C317 | E |
| Cameroon | C331 | E |
| Cameroon | C339 | E |
| Cameroon | C344 | E |
| Cameroon | C350 | T |
| Cameroon | C354 | T |
| Cameroon | C496 | E |
| Cameroon | C541 | T |
| Cameroon | C547 | T |
| Cameroon | C575 | T |
| BPRC | CAROLINA | W |
| BPRC | CINDY | W |
| BPRC | DEBBIE | W |
| BPRC | FRITS | W |
| BPRC | LADY | W |
| BPRC | LIESBETT | W |
| BPRC | LOUISE | W |
| BPRC | PEARL | W |
| BPRC | RENEE | W |
| BPRC | SHERRY | W |
| BPRC | SONJA | W |
| BPRC | SUSIE | W |
| BPRC | TOETIE | W |
| BPRC | WODKA | W |
| BPRC | YOLANDA | W |

BPRC = Biomedical Primate Research Centre, The Netherlands

mtDNA classification: T, *P. t. troglodytes*; E, *P. t. ellioti*; W, ‘Western’ i.e. *P. t. verus.*
