# Supplementary material for: Genomic Tools for Evolution and Conservation in the Chimpanzee: Pan troglodytes ellioti Is a Genetically Distinct Population
Source: PLoS Genet. 2012 Mar 1;8(3):e1002504. doi: 10.1371/journal.pgen.1002504 (PMC3291532; doi:10.1371/journal.pgen.1002504)
Supplement: Table S2 — Amplification Targets. (DOC) [file pgen.1002504.s006.doc]

### Table S2: Amplification Targets

| gene | target region | bp amplified | incl bp coding seq | GenBank accession |
| --- | --- | --- | --- | --- |
| CCR5 | exon 4 | 1054 | 1054 | NM_000579 |
| SDF | 3’UTR | 926 | 0 | AL713778 |
| CXCR4 | exon 2 | 1081 | 1081 | AF025375 |
| CX_3_CR1 | exon 2 | 1193 | 1193 | HSU20350 |
| RANTES | promoter | 988 | 0 | AB023652 |
| CCR2 | exon 2 | 1096 | 1096 | NM_000648 |
| SEC22L3 | intron 6, exon 7 | 1017 | 545 | NC_000003 |
| ZNF445 | exon 6 | 1077 | 1077 | NC_000003 |
| PTPN23 | exon 20 | 1016 | 1016 | NM_015466 |
| CCRL2 | exon 2 | 1035 | 1035 | NM_003965 |
| MC1R | exon 1 | 1122 | 1122 | NM_002386 |
| β-globin cluster | R/T | 1113 | 0 | AF339406 |
| mtDNA | HV-I | 534 | 0 | AJ586556 |
