# Supplementary material for: Genomic Tools for Evolution and Conservation in the Chimpanzee: Pan troglodytes ellioti Is a Genetically Distinct Population
Source: PLoS Genet. 2012 Mar 1;8(3):e1002504. doi: 10.1371/journal.pgen.1002504 (PMC3291532; doi:10.1371/journal.pgen.1002504)
Supplement: Table S3 — PCR and Sequencing Primers. PCR primers are in bold. All primers were used for sequencing. (DOC) [file pgen.1002504.s007.doc]

### Table S3 PCR and Sequencing Primers

PCR primers are in bold. All primers were used for sequencing.

| **Primer** | **Sequence (5’-3’)** |
| --- | --- |
| **CCR5 1F** | **ATTTATGCACAGGGTGGAAC** |
| **CCR5 1R** | **TTGAGTCCGTGTCACAAGC** |
| CCR5 2F | GTGTTTGCATCTCTCCCAGG |
| CCR5 2R | ACAGCCACCACCCAAGTGA |
| CCR5 3F | GGCACAGGGCTGTGAGGCT |
| **CCR2 2F** | **ACGCATTTCCCCAGTACATC** |
| **CCR2 2R** | **GAGACTTCCTGCTCCCCAGT*** |
| CCR2 3F | CACAGGGCTGTATCACATCG |
| CCR2 4F | CATTGTCATTCTCCTGAACACC |
| CCR2 4R | GGAAGGCGTGTTTGTTGAAG |
| CCR2 5R | GGTGTTCAGGAGAATGACAATG |
| CCR2 6R | ATGAAGAAGATTCCGCCAAA |
| **CX3CR 2F** | **TTCTTCCTGCCTGCCTACAT** |
| **CX3CR 2R** | **GACACAAGGCTTTGGGATTC** |
| CX3CR 3F | CTTCATCGGCTTTTTGGAAG |
| CX3CR 3R | CTTCCAAAAAGCCGATGAAG |
| CX3CR 4F | GGTGGTCATCGTGTTTTTCC |
| CX3CR 4R | GAAAAACACGATGACCACCA |
| **CXCR4 1F** | **CATTCCTTTGCCTCTTTTGC** |
| **CXCR4 1R** | **TCTTTTACATCTGTGTTAGCTGGAG** |
| CXCR4 2F | ACAGCAGTGTCCTCATCCTG |
| CXCR4 2R | AAGGCCAGGATGAGGACACT |
| CXCR4 3F | GCCCTCAAGACCACAGTCAT |
| CXCR4 3R | CCAGGATGAGGATGACTGTG |
| **RANTES 1R** | **TGGTGGTCAAGACCAGGAC** |
| **RANTES 3F** | **ACGCGCTAAGACAGTGGAAT** |
| RANTES 4F | CACCTCCTTTGGGGACTGTA |
| RANTES 4R | TACAGTCCCCAAAGGAGGTG |
| RANTES 5F | CGAATTTCCGGAGGCTATTT |
| RANTES 5R | AAATAGCCTCCGGAAATTCG |
| **SDF 1R** | **ATGAGGGCTGGGTCTCACT** |
| **SDF 2F** | **CCGTCGTATGACCACTCTGA** |
| SDF 4F | CAACAGGAGGTTCAAGATGTGA |
| SDF 4R | TCACATCTTGAACCTCCTGTTG |
| SDF 5F | GTGCAGGTGGGGAGACTG |
| SDF 5R | CAGTCTCCCCACCTGCAC |
| **SEC 1F** | **TGGCTTTCTCTCACCAGCTT** |
| **SEC 1R** | **TGGTTCCTTGGAGACAACTCT** |
| SEC 3F | GTGGGAGGTGAATTTTGCAT |
| SEC 3R | GCAAAATTCACCTCCCACAG |
| SEC 4F | TTCCATCCTTTTCAGCCAAC |
| SEC 4R | GTTGGCTGAAAAGGATGGAA |
| **ZNF 1F** | **TGCGACAAGTGTTTCTGAGG** |
| **ZNF 1R** | **CGGTGGTCAACGAGAGTTTT** |
| ZNF 2F | TGGGCAGAGTCTTCACACAG |
| ZNF 2R | AATGACACCCCACTGTGTGA |
| ZNF 3F | GCAGGAAATCCTTCCACTGT |
| ZNF 3R | TGGAAGGATTTCCTGCACTG |
| **PTPN 5F** | **CCCACGCCTTACACCTACC** |
| **PTPN 6R** | **CATGAGCCAGAAGTCAGCAG** |
| PTPN 7F | AGCCCCTCTTACCATTCGAG |
| PTPN 7R | GTAGAAGGGGCAGGACCATA |
| PTPN 8F | ATGTGGGAGCTCTGGACACT |
| PTPN 8R | AGTGTCCAGAGCTCCCACAT |
| **CCRL2 1F** | **TGGGCCTGTACAGTGAGACA** |
| **CCRL2 1R** | **GGCTCTGAGCAAATGCTACC** |
| CCRL2 2F | AAGGTTCAGGGAGCAGAGGT |
| CCRL2 2R | ACCTCTGCTCCCTGAACCTT |
| CCRL2 3F | TAGCATCCACCAAATGCAAG |
| CCRL2 3R | CTTGCATTTGGTGGATGCTA |
| **MC 2F** | **GCAGCACCATGAACTAAGCA** |
| **MC 2R** | **GGTCACACAGGAACCAGACC** |
| MC 3F | TGCAGCAGGTGGACAATG |
| MC 3R | ATGACATTGTCCACCTGCTG |
| MC 4F | TGTCACCCTCACCATCCTG |
| MC 4R | CAGGATGGTGAGGGTGACA |
| **RT 4F** | **TGTGAATTTTGTAGAAGGATATAACCCATAT** |
| **RT 6R** | **GGATTGCATTGAGAGGATTTG** |
| SEQRT 3F | CCTCTGACCTCCAACCTCAG |
| SEQRT 1R | CAGAGTTGGGAATTGGGATT |
| SEQRT 2F | GCAGGCAGATCACTTGAGGT |
| SEQRT 2R | TCCTGACCTCAAGTGATCTGC |
| **L15996** | **CTCCACCATTAGCACCCAAAGC** |
| **H16498** | **CCTGAAGTAGGAACCAGATG** |
