# Supplementary material for: Genomic Tools for Evolution and Conservation in the Chimpanzee: Pan troglodytes ellioti Is a Genetically Distinct Population
Source: PLoS Genet. 2012 Mar 1;8(3):e1002504. doi: 10.1371/journal.pgen.1002504 (PMC3291532; doi:10.1371/journal.pgen.1002504)
Supplement: Table S4 — Highly Differentiated SNPs. For each population, SNPs with highest frequency difference between chimpanzees in that population cluster and the other two clusters. Bold SNPs are in the minimal panel of 10 markers used in Figure 2b to reproduce the original clustering pattern; SNPs from re-sequencing are underlined. (DOC) [file pgen.1002504.s008.doc]

### Table S4 Highly Differentiated SNPs

For each population, SNPs with highest frequency difference between chimpanzees in that population cluster and the other two clusters. Bold SNPs are in the minimal panel of 10 markers used in Figure 2(b) to reproduce the original clustering pattern; SNPs from re-sequencing are underlined.

|  | SNP name | | dbSNP | | frequency difference |f*_x_* - (f_y_+f*_z_*)/2| | |  |
| --- | --- | --- | --- | --- | --- | --- | --- |
| *P. t. ellioti* |  | |  | |  | |  |
|  | **MC1R-01** | | **NA** | | **0.660** | |  |
|  | **THE-02-L22** | | **rs24982023** | | **0.633** | |  |
|  | **L2-13-R13** | | **rs26272363** | | **0.629** | |  |
|  | **L2-18-L13** | | **rs26731159** | | **0.601** | |  |
|  | PTPN23-01 | | NA | | 0.586 | |  |
|  | THE-14-R12 | | rs25310572 | | 0.585 | |  |
|  | L2-03-L03 | | rs25073049 | | 0.582 | |  |
|  | THE-01-R11 | | rs26149979 | | 0.551 | |  |
|  | L2-21-R14 | | rs26106336 | | 0.539 | |  |
|  | THE-26-R08 | | rs26225579 | | 0.524 | |  |
|  | THE-39-R17 | | rs25711712 | | 0.517 | |  |
|  | THE-03-L04 | | rs25732989 | | 0.508 | |  |
|  |  | |  | |  | |  |
| *P. t. verus* |  | |  | |  | |  |
|  | **THE-01-R08** | | **rs26684235** | | **0.988** | |  |
|  | **L2-18-L19** | | **rs24968101** | | **0.967** | |  |
|  | **L2-21-R03** | | **rs25430328** | | **0.939** | |  |
|  | THE-03-L08 | | rs25734025 | | 0.908 | |  |
|  | THE-02-L08 | | rs26584808 | | 0.904 | |  |
|  | THE-39-L09 | | rs26125238 | | 0.904 | |  |
|  | L2-13-R21 | | rs24975117 | | 0.902 | |  |
|  | THE-59-R03 | | rs25196242 | | 0.900 | |  |
|  | THE-43-R09 | | rs25111879 | | 0.867 | |  |
|  | THE-24-L01 | | rs25707792 | | 0.867 | |  |
|  | THE-26-L10 | | rs26205640 | | 0.862 | |  |
|  | THE-48-L11 | | rs26192085 | | 0.815 | |  |
|  |  | |  | |  | |  |
| *P. t. troglodytes* | |  | |  | |  | |
|  | **THE-34-R06** | | **rs25346157** | | **0.866** | |  |
|  | **L2-11-L10** | | **rs26089887** | | **0.734** | |  |
|  | **L2-13-L14** | | **rs24986665** | | **0.729** | |  |
|  | L2-12-R05 | | rs25196439 | | 0.682 | |  |
|  | L2-21-R16 | | rs25419851 | | 0.660 | |  |
|  | THE-43-R05 | | rs25220991 | | 0.612 | |  |
|  | THE-15-L06 | | rs25016979 | | 0.596 | |  |
|  | L2-03-L06 | | rs25120739 | | 0.585 | |  |
|  | THE-23-L07 | | rs25027098 | | 0.575 | |  |
|  | THE-48-L11 | | rs26192085 | | 0.572 | |  |
|  | THE-39-R19 | | rs26170406 | | 0.559 | |  |
|  | L2-18-L14 | | rs26673756 | | 0.550 | |  |
